# Telephone-based lifestyle education to prevent gestational diabetes in high-risk Iranian women: a randomized trial

**DOI:** 10.1186/s12884-026-08830-x

**Published:** 2026-02-20

**Authors:** Zahra Gorjian, Nahal Nabhani, Atefeh Zahedi

**Affiliations:** 1 Department of Medical-Surgical Nursing, Abadan University of Medical Sciences, Abadan, Iran; 2 Student Research Committee, Abadan University of Medical Sciences, Abadan, Iran; 3 Asadabad School of Medical Sciences, Asadabad, Iran

**Keywords:** Gestational Diabetes Mellitus, Telemedicine, Lifestyle Intervention, Pregnancy, Disease Prevention

## Abstract

**Background:**

Gestational diabetes mellitus (GDM) is a common metabolic disorder during pregnancy, associated with increased maternal and neonatal complications. This study aimed to evaluate the effectiveness of telephone-based lifestyle education in preventing GDM among high-risk Iranian women.

**Methods:**

A parallel randomized clinical trial was conducted from October 2024 to June 2025 at primary healthcare centers. Among 1,000 pregnant women assessed for eligibility, 180 high-risk participants with normal baseline blood glucose were selected. Of these, 170 participants were randomly assigned to the intervention (*n* = 85) and control (*n* = 85) groups. Five participants from the intervention group and seven from the control group were lost to follow-up, leaving 80 participants in the intervention group and 78 in the control group who completed the study. The intervention group received structured telephone counseling on healthy nutrition, physical activity, weight management, and blood glucose monitoring, while the control group received routine prenatal care. This intervention was delivered via telephone and consisted of seven structured counseling sessions. Data were collected using a checklist of structured questions designed to assess adherence to self-care behaviors, including healthy diet, physical activity, and blood glucose monitoring. The checklist also included questions on demographic information and adherence to the interventions. The primary outcome was GDM incidence at 28 weeks of gestation. Secondary outcomes included changes in BMI and adherence to self-care behaviors. Statistical analysis was performed using IBM SPSS Statistics version 21.

Data were analyzed using chi-square tests for categorical variables and independent t-tests for continuous variables. Multivariable logistic regression was used for the primary outcome. A p-value < 0.05 was considered statistically significant.

**Results:**

At 28 weeks, GDM occurred in 10% of the intervention group compared to 24.4% of controls (*p* = 0.018). After adjusting for confounders, telephone education was associated with a significant reduction in GDM risk (adjusted OR = 0.37; 95% CI: 0.15–0.88; *p* = 0.025). BMI increase was lower in the intervention group (0.6 ± 1.1 vs. 1.3 ± 1.4, *p* = 0.012). Adherence to a healthy diet, regular physical activity, and blood glucose monitoring in the intervention group was 75%, 68%, and 62%, respectively, significantly higher than the control group.

**Conclusion:**

Telephone-based lifestyle education is an effective, accessible, and cost-efficient strategy to prevent GDM in high-risk women and can improve self-care behaviors and weight management during pregnancy.

**Trial registration:**

IRCT registration number: IRCT2017052115995N2 Registration date: 2017-09-22.

**Supplementary Information:**

The online version contains supplementary material available at 10.1186/s12884-026-08830-x.

## Background

Gestational diabetes mellitus (GDM), defined as glucose intolerance first diagnosed during pregnancy, arises from hormonal changes, such as increased human placental lactogen and progesterone, which elevate maternal insulin resistance [1]. When pancreatic β-cell function cannot compensate, hyperglycemia develops [2]. GDM affects a substantial number of pregnancies worldwide, with prevalence estimates ranging from 1% to 14%. In the Eastern Mediterranean Region, the pooled prevalence is around 11% (95% CI: 9–14%), while in Iran, it varies from 1.3% to 18.6%, particularly among women with known risk factors [3–5]. The most common risk factors in this population include obesity, family history of diabetes, advanced maternal age, and a previous history of GDM or macrosomic birth.

In Iran, challenges in accessing specialized prenatal care, along with cultural and social factors such as limited awareness of self-care practices and traditional dietary preferences, increase the difficulty of preventing GDM ([4–5]). The consequences of GDM are significant, impacting both maternal and neonatal health. These include preeclampsia, macrosomia, birth trauma, polyhydramnios, preterm delivery, neonatal metabolic disturbances, and a higher long-term risk of type 2 diabetes for both mother and child [6–8].

In recent years, telephone-based education has emerged as a low-cost, scalable, and culturally adaptable solution for health interventions. This method is particularly beneficial in low-resource settings where face-to-face consultations may be challenging due to geographical, financial, or time constraints. Unlike smartphone applications, which require advanced digital literacy and stable internet access, telephone interventions are accessible to a wider population, including those in remote areas. Moreover, telephone-based interventions offer consistent, personalized support, leading to improved adherence to health behaviors. The cost-effectiveness and ability to reach a large number of people with minimal resources make this approach an ideal solution for preventing conditions like gestational diabetes mellitus (GDM) in populations with limited access to specialized care ([9–10]). Telephone-based education has demonstrated benefits in managing chronic diseases and supporting postpartum lifestyle changes for women with a history of GDM (9(. Yet, its preventive impact during pregnancy remains underexplored, with challenges such as limited adherence due to time constraints and variable effectiveness across culturally diverse populations [11–14].

Therefore, the main objective of this study was to evaluate the effectiveness of a structured telephone-based lifestyle education program in preventing gestational diabetes mellitus (GDM) among high-risk pregnant women in Iran. Additionally, the study aimed to assess the program’s impact on weight management and adherence to self-care behaviors.

### Research Question

The main research question of this study is: Can a structured telephone-based lifestyle education program reduce the incidence of gestational diabetes mellitus in high-risk pregnant women compared to standard prenatal care?

### Hypothesis

We hypothesize that a structured, telephone-based intervention will significantly reduce the incidence of GDM in high-risk pregnant women compared to those who receive standard prenatal care.

## Methods

### Study design and setting

This parallel-group randomized controlled trial (RCT) was conducted between 2024 and 2025 among pregnant women attending 25 urban and rural primary health care centers affiliated with Abadan University of Medical Sciences. These centers are comprehensive community health facilities that provide a broad range of preventive and clinical services, including child vaccination, family health, and management of chronic conditions. Each center also has a dedicated maternal and child health unit responsible for antenatal, intrapartum, and postpartum care, from which eligible pregnant women were recruited. The study was approved by the Ethics Committee of Abadan University of Medical Sciences (IR.ABADANUMS.REC.1396.201), and all participants provided written informed consent prior to enrollment, confirming that they were voluntarily participating in a research study. However, they were not informed of their group allocation to minimize bias. The trial was registered at the Iranian Registry of Clinical Trials (IRCT) under the registration number IRCT2017052115995N2.

This study adheres to the CONSORT 2025 guidelines for reporting randomized controlled trials.

Data collection began in October 2024 and concluded in June 2025, covering both the intervention period and follow-up assessments at 28 weeks of gestation. All institutional and ethical approvals were in place, and the study was conducted in accordance with CONSORT guidelines.

In this study, gestational diabetes screening was performed for all participants at 28 weeks, regardless of their risk factors, as part of the research protocol. This screening was conducted at primary health care centers as part of the routine clinical services available to all participants. However, routine GDM screening is not mandatory for all pregnant women in Iran and is generally performed for women with known risk factors, such as obesity, previous GDM, or advanced maternal age.

No patient or public involvement was included in the design, conduct, or reporting of this trial.

There were no changes to eligibility criteria, interventions, or outcomes after trial initiation.

### Participants and Screening

A total of 1,000 pregnant women presenting for their first antenatal visit were initially screened for eligibility. Screening included assessment of demographic data, obstetric history, and known risk factors for gestational diabetes mellitus (GDM). Women with at least two major risk factors—such as body mass index (BMI) ≥ 30 kg/m², first-degree family history of diabetes, prior history of GDM, or previous delivery of a macrosomic infant (> 4 kg)—were identified as high-risk, as defined by Kautzky-Willer et al., 2023 [15].

Among these, 180 women met the high-risk criteria and underwent baseline laboratory glucose testing to exclude pre-existing diabetes or impaired glucose tolerance. Fasting blood sugar (FBS) and 2-hour postprandial glucose (2hPP) were measured according to standard protocols. Ten women were excluded due to abnormal glucose levels indicative of impaired glucose metabolism or undiagnosed diabetes (FBS > 92 mg/dL or 2hPP ≥ 140 mg/dL).

**Fig. 1:**
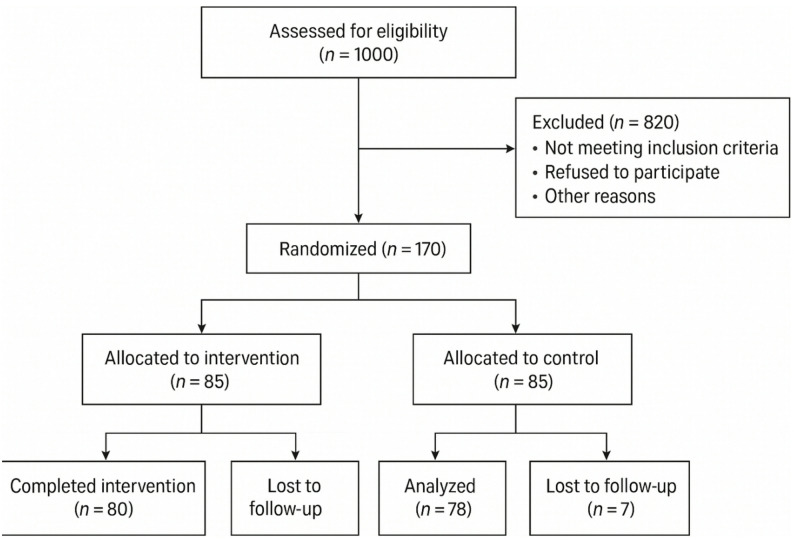
Flow diagram of participant recruitment, screening, eligibility, and randomization

Out of the remaining 170 eligible participants with normal baseline glucose levels, all were enrolled by trained research staff at the primary healthcare centers. The randomization process was conducted by an independent statistician who used block randomization to assign participants to either the intervention group or the control group. The allocation sequence was concealed from the research team until assignment. A total of 85 participants were allocated to the intervention group, and 85 to the control group. The sample size was initially calculated based on an effect size of 0.5, a significance level of 0.05, and 80% power, resulting in a minimum of 63 participants per group (total 126). To account for potential dropouts and increase power, 170 participants were ultimately recruited. The flow of participants through the trial is shown in Fig. 1.

### Inclusion and Exclusion Criteria

#### Inclusion criteria were

Age 18–35 years, gestational age under 12 weeks at recruitment, presence of at least two major GDM risk factors, normal baseline glucose tests, ability to read and write (minimum education level), no history of drug addiction, and reliable access to a telephone for follow-up.

#### Exclusion criteria included

Participants with complications such as preeclampsia, preterm labor, or other significant pregnancy-related conditions (e.g., placental abnormalities, gestational hypertension) were excluded after randomization. These exclusions were necessary to ensure that the study outcomes were not confounded by conditions that could independently affect the results.

### Randomization and Allocation

The 170 eligible participants were randomly assigned in a 1:1 ratio to the intervention group (*n* = 85) or the control group (*n* = 85) using block randomization with a computer-generated sequence. The block size was 4, and randomization was performed by a statistician independent of the study team. Blocking was stratified based on education level and BMI to ensure balanced distribution of these variables across groups and minimize bias.

Participants in both the intervention and control groups were not aware of their group allocation. Laboratory personnel assessing glucose outcomes were blinded to group allocation. Due to the nature of the intervention, care providers were not blinded.

### Intervention

Participants in the intervention group received structured telephone-based lifestyle education in addition to standard prenatal care. The intervention was delivered by trained midwives and nurses under the supervision of a faculty member in maternal health. No dietitians were directly involved in either the intervention or the standard care process. The structured telephone counseling focused on healthy nutrition, physical activity, weight management, and blood glucose monitoring.

However, no glucometers were provided to participants, and no education on blood glucose monitoring was given. Instead, gestational diabetes screening was performed for all participants at 28 weeks of pregnancy as part of routine prenatal care at primary health care centers.

Each counseling session complemented the routine in-person care that all pregnant women received at primary health care centers, ensuring consistency between both care modalities. Approximately every 10 days until the 28th week of gestation, personalized telephone counseling sessions were delivered, focusing on promoting healthy dietary patterns (such as emphasizing low-glycemic index foods, balanced portions of fruits, vegetables, whole grains, and lean proteins while limiting sugary or processed items), safe physical activity (including moderate exercises like daily 30-minute walks, prenatal yoga, or light strength training adapted to individual fitness levels and pregnancy stage), recognition of GDM warning signs (e.g., excessive thirst, frequent urination, or unexplained fatigue), and weight management through practical daily self-care strategies (like tracking weekly weight gain and adjusting habits accordingly). Educational materials were drawn from Koivusalo et al. 2016 [16], and standard obstetric references (e.g., Williams Obstetrics), with content customized to cultural preferences in Iran, such as incorporating traditional foods in a healthy way.

A total of seven sessions were conducted for the intervention group, with each call lasting 10–15 min. Participants who did not follow the recommendations were not given extra time or additional sessions beyond the standard seven calls. Traditional foods were incorporated in a healthy way by providing simple advice on modifying traditional recipes to reduce added sugars, fats, and processed ingredients. For example, participants were encouraged to replace sugary snacks with low-glycemic alternatives, such as substituting fruit-based desserts or using healthier cooking methods like grilling or steaming instead of frying.

Additionally, traditional Iranian dishes, such as stews and rice dishes, were adapted to include healthier ingredients and cooking techniques. For instance, participants were encouraged to use whole grains instead of white rice, reduce the amount of oil in stews, and include more vegetables in dishes like khoresh and pilaf.

The region where this study was conducted is particularly known for its date production, and local people often use dates to prepare various traditional desserts. These desserts typically contain high levels of sugar, fat, and additives. In the intervention, recommendations were made to use dates in controlled amounts and to incorporate healthier alternatives, in order to reduce excessive sugar and fat intake.

During each follow-up call, adherence was evaluated through brief self-reports from participants, and customized feedback was provided— for instance, if a participant reported low activity levels, suggestions might include starting with short home-based routines or involving family support. A detailed schedule of the telephone-based lifestyle education sessions, including content topics, session durations, and learning objectives, is presented in Table 1.

**Table 1:** Telephone-Based Lifestyle Education Session Schedule

Session No.	Topic/Content	Approx. Duration	Learning Goals/Key Points	Implementation Strategy	
1	Introduction to the Program & Setting Goals	15 min	Welcome participants, explain program structure, set personal health goals (e.g., maintaining healthy weight gain), and motivate adherence through discussing GDM risks and benefits of self-care.	Interactive telephone discussion on goal setting, use of visual aids to explain GDM risks, and personal goal-setting activity for participants to identify their health goals.	
2	Healthy Eating & Weight Control	15 min	Guide on balanced nutrition, emphasizing low-glycemic foods (e.g., lentils, whole-grain bread, yogurt) and portion control, incorporating Iranian staples like sabzi (a traditional Iranian herb-based dish) or low-sugar fruits. Discuss tracking weekly weight gain.	Telephone-based dietary education through visual examples, providing simple recipe modifications, and discussing culturally relevant food choices. Participants are encouraged to keep a food diary to track their progress.	
3	Safe Physical Activity	15 min	Encourage 30-min daily activities like walking or prenatal yoga, tailored to pregnancy stage and fitness level. Provide examples (e.g., short home-based stretches) and address safety concerns.	Telephone instruction on exercises suitable for pregnancy, with step-by-step guidance. Participants are encouraged to perform exercises and log their daily activities for motivation.	
4	Blood Sugar Monitoring & Health Awareness	10 min	recognize GDM warning signs (e.g., excessive thirst), and maintain regular monitoring. Discuss importance of consistent check-ups.	Interactive telephone demonstration on how to use a glucometer and role-play scenarios for identifying GDM warning signs. Participants are educated on tracking their blood sugar levels and setting up regular check-ups with healthcare providers.	
5	Managing Stress & Improving Sleep	10 min	Share simple stress-reduction techniques (e.g., deep breathing, mindfulness) and tips for better sleep (e.g., avoiding screens before bed). Tailor advice to participants’ daily routines.	Guided telephone-based mindfulness exercises to reduce stress, sharing relaxation techniques. Participants are encouraged to apply these techniques before bedtime and share their experiences during follow-up calls.	
6	Review & Personalized Feedback	15 min	Discuss progress, address challenges (e.g., difficulty following diet), and provide tailored suggestions (e.g., swapping high-sugar snacks with nuts). Reinforce key concepts from prior sessions.	Personalized telephone feedback based on self-reports. Participants are encouraged to share their challenges, receive motivational support, and discuss how to overcome obstacles through individualized coaching.	
7	Planning for Continued Health	15 min	Help create a sustainable self-care plan, including realistic diet and exercise routines. Encourage family involvement and long-term commitment to healthy habits.	Telephone-based goal-setting for future health with practical steps. Participants are encouraged to involve family members in their health goals and provided with practical tips for maintaining healthy habits after the program ends.	

"Participants in the intervention group received a total of seven structured telephone counseling sessions, conducted approximately every 10 days until 28 weeks of gestation. Each call lasted 10–15 min and included feedback on participants’ progress regarding diet, physical activity, and weight management goals."

### Training of intervention staff and fidelity monitoring

All intervention staff were trained using a standardized script that included detailed guidelines for conducting telephone counseling on healthy nutrition, physical activity, weight management, and blood glucose monitoring. The training sessions were held over a period of two days, and staff were provided with feedback to ensure consistency and accuracy in the delivery of the intervention.

Fidelity monitoring was carried out through random audits of intervention calls. Supervisors listened to 10% of the calls to ensure that staff adhered to the prescribed protocol and provided the correct information. Any deviations from the protocol were addressed immediately in subsequent training sessions. Additionally, regular meetings were held to discuss challenges and improve the intervention delivery process.

### Control group

Participants in the control group received routine prenatal care provided at primary healthcare centers in Iran. Standard care was offered in person at all urban and rural health centers to all pregnant women, including both study groups.

It followed the national maternal health protocol, which includes regular antenatal visits with weight and BMI monitoring, blood pressure monitoring, fetal growth assessment, and general counseling on nutrition and physical activity. Basic health education on pregnancy self-care was also provided.

No specific or specialized program for preventing gestational diabetes mellitus (GDM) was offered beyond standard care. Participants in the control group did not receive any telephone calls during the study period. They only attended routine in-person visits at the primary health care centers, where demographic and baseline information were collected during the first visit, and laboratory testing for GDM diagnosis was conducted at 28 weeks of gestation. No telephone-based monitoring or counseling was provided for this group.

### Outcome measures

The main outcome was the incidence of GDM at 28 weeks, diagnosed based on a 50 g Glucose Challenge Test (GCT) followed by a 75 g Oral Glucose Tolerance Test (OGTT) if indicated. Secondary outcomes included changes in BMI from baseline to 28 weeks and adherence to self-care behaviors such as diet, physical activity, and glucose monitoring. Self-care adherence was assessed through structured questions asked during telephone follow-ups, acknowledging the potential for self-report bias.

### Laboratory procedures

Blood glucose measurements followed standard procedures. Normal baseline glucose was defined as FBS ≤ 92 mg/dL and 2hPP < 140 mg/dL. At 28 weeks, the GCT was performed by administering 50 g of glucose orally (non-fasting), with plasma glucose measured after 1 h; a value ≥ 130 mg/dL was considered abnormal. Women with abnormal or borderline GCT underwent a 75 g OGTT, with Diagnosis of GDM was based on the OGTT criteria (2-hour glucose ≥ 135 mg/dL), following the national guideline of the Iranian Ministry of Health (2017), which recommends this threshold for screening purposes. To ensure comparability with international standards, additional sensitivity analyses were also conducted using cut-offs of ≥ 140 mg/dL (Carpenter–Coustan) and ≥ 153 mg/dL (IADPSG) [17].

At 28 weeks, the GDM screening was performed by administering the 50 g Glucose Challenge Test (GCT), followed by a 75 g Oral Glucose Tolerance Test (OGTT) if indicated. This screening was done for all participants as part of the study and was conducted at primary health care centers. However, in routine practice, this screening is typically performed for women with known risk factors and not routinely for all pregnant women.

### Data collection

Baseline demographic and clinical data were collected at enrollment, and during follow-up, adherence to dietary, physical activity, and glucose monitoring recommendations was assessed using structured questions developed by the research team. Anthropometric measurements (weight and height) were taken in person at baseline using calibrated instruments, and weight measurements were self-reported during follow-up telephone calls. Laboratory measurements were documented at baseline and at 28 weeks of gestation. Adherence to self-care behaviors was assessed using structured questions developed by the research team during telephone follow-ups. These questions were designed based on relevant literature and clinical guidelines, and they were reviewed for clarity and cultural appropriateness. However, they were not formally pretested or validated.

### Statistical analysis

Data were analyzed using IBM SPSS Statistics version 21. Continuous variables were expressed as means ± standard deviations, and categorical variables as frequencies and percentages. Baseline characteristics were compared between groups using independent t-tests for continuous variables and chi-square tests for categorical variables.

The primary outcome, incidence of GDM, was compared between groups using chi-square tests. To adjust for potential confounders, multivariable logistic regression analysis was performed with GDM incidence as the dependent variable and group assignment (intervention vs. control) as the primary independent variable. Covariates included in the model were age, baseline BMI, education level, employment status, and family history of diabetes, selected based on clinical relevance and prior literature. Missing data were handled using multiple imputation under the assumption of missing at random (MAR), and results were pooled across all imputed datasets according to Rubin’s rules.

An intention-to-treat (ITT) analysis was conducted, including all participants who were randomized into the study, even if they dropped out or had missing data. This approach ensured that all participants were considered in the analysis, regardless of whether they completed the study.

Model assumptions and goodness-of-fit were assessed using the Hosmer–Lemeshow test, with a non-significant result (*p* > 0.05) indicating an adequate fit. Multicollinearity among covariates was evaluated using Variance Inflation Factors (VIF), with values below 5 suggesting no significant multicollinearity. Adjusted odds ratios (aORs) with 95% confidence intervals (CIs) and corresponding p-values were reported for all covariates. Secondary outcomes, including changes in BMI and adherence rates to self-care behaviors, were compared between groups using independent t-tests or chi-square tests, depending on the type of variable. A p-value < 0.05 was considered statistically significant.

## Results

### Participant flow and baseline characteristics

Of the 1,000 pregnant women initially screened, 180 met the high-risk criteria for GDM and underwent baseline glucose testing. Ten women were excluded due to abnormal results (fasting blood sugar > 92 mg/dL or 2-hour postprandial glucose ≥ 140 mg/dL), leaving 170 eligible participants who were randomized into the intervention (*n* = 85) and control (*n* = 85) groups.

Eligible women were randomly assigned in equal numbers to the intervention group (*n* = 85) and the control group (*n* = 85) using block randomization. During follow-up, 5 participants from the intervention group were lost due to missed telephone contacts (*n* = 3) and pregnancy complications unrelated to GDM, such as preeclampsia or preterm labor (*n* = 2). Similarly, 7 participants from the control group discontinued the study for reasons including missed telephone contacts (*n* = 4) and non-GDM pregnancy complications (*n* = 3). Thus, 80 participants in the intervention group and 78 in the control group completed the study.

Baseline demographic and clinical characteristics before attrition were comparable between groups (all *p* > 0.05). Importantly, baseline characteristics of participants who completed the study also remained comparable after attrition (all *p* > 0.05), indicating that loss to follow-up did not substantially affect group equivalence (Tables 2 and 3).

**Table 2:** Baseline characteristics before attrition (*n* = 85 per group)

Characteristic	Intervention (*n* = 85)	Control (*n* = 85)	*p*-value
Age (years), mean ± SD	28.4 ± 4.2	28.7 ± 4.1	*p* > 0.05
BMI (kg/m²), mean ± SD	32.1 ± 3.5	31.8 ± 3.7	*p* > 0.05
Education level ≥ 5th grade, n (%)	66 (77.6%)	64 (75.3%)	*p* > 0.05
Employed, n (%)	43 (50.6%)	41 (48.2%)	*p* > 0.05
Family history of diabetes, n (%)	46 (54.1%)	47 (55.3%)	*p* > 0.05
Prior GDM history, n (%)	11 (12.9%)	10 (11.8%)	*p* > 0.05
Prior macrosomic infant, n (%)	14 (16.5%)	15 (17.6%)	*p* > 0.05

**Table 3:** Baseline characteristics after attrition (Intervention *n* = 80, Control *n* = 78)

Characteristic	Intervention (*n* = 80)	Control (*n* = 78)	*p*-value
Age (years), mean ± SD	28.3 ± 4.1	28.6 ± 4.0	*p* > 0.05
BMI (kg/m²), mean ± SD	32.0 ± 3.4	31.7 ± 3.6	*p* > 0.05
Education level ≥ 5th grade, n (%)	62 (77.5%)	59 (75.6%)	*p* > 0.05
Employed, n (%)	40 (50.0%)	38 (48.7%)	*p* > 0.05
Family history of diabetes, n (%)	43 (53.8%)	42 (53.8%)	*p* > 0.05
Prior GDM history, n (%)	10 (12.5%)	9 (11.5%)	*p* > 0.05
Prior macrosomic infant, n (%)	13 (16.3%)	13 (16.7%)	*p* > 0.05

### Primary Outcome: Incidence of GDM

At 28 weeks’ gestation, the incidence of GDM was significantly lower in the intervention group compared to the control group: 8 cases (10.0%) versus 19 cases (24.4%), respectively (*p* = 0.02) (Table 4).

Multivariable logistic regression adjusting for age, baseline BMI, education, employment status, and family history of diabetes confirmed that telephone-based self-care education was associated with a significantly reduced risk of developing GDM (adjusted Odds Ratio [aOR] = 0.37; 95% Confidence Interval [CI]: 0.15–0.88; *p* = 0.03). Model fit was assessed using the Hosmer–Lemeshow goodness-of-fit test, indicating an acceptable fit, and multicollinearity among covariates was evaluated using variance inflation factors (VIF), which showed no significant multicollinearity.

**Table 4:** Primary Outcome — Incidence of Gestational Diabetes Mellitus (GDM) at 28 Weeks

Outcome	Intervention (*n* = 80)	Control (*n* = 78)	*p*-value
GDM cases, n (%)	8 (10.0%)	19 (24.4%)	0.02

*Values are presented as frequencies (%). *p*-values are based on chi-square tests (crude comparison)

Adjusted odds ratios (aOR) were obtained using multivariable logistic regression controlling for age, baseline BMI, education level, employment status, and family history of diabetes

### Secondary Outcomes

#### Change in BMI

The mean BMI increase from baseline to 28 weeks was significantly lower in the intervention group compared to controls (0.6 ± 1.1 kg/m² vs. 1.3 ± 1.4 kg/m², *p* = 0.01) (Table 5).

**Table 5:** Secondary Outcome — Change in BMI from Baseline to 28 Weeks

Outcome	Intervention (*n* = 80)	Control (*n* = 78)	*p*-value
BMI change (kg/m²)	0.6 ± 1.1	1.3 ± 1.4	0.01

*Values are presented as mean ± SD. Independent t-test was used to compare groups; results are unadjusted (crude comparison)

### Adherence to Self-Care Behaviors

Self-reported adherence rates to key self-care behaviors were significantly higher in the intervention group. For example, 75% of the intervention group reported following dietary recommendations, compared to 50% in the control group (*p* < 0.001). Similarly, 68% engaged in regular physical activity versus 45% in controls (*p* < 0.001), and 62% performed blood glucose monitoring compared to 44% of controls (*p* = 0.02) (Table 6).

**Table 6:** Adherence to recommended self-care behaviors among intervention and control groups at 28 weeks’ gestation

Behavior	Intervention (*n* = 80)	Control (*n* = 78)	*p*-value
Healthy diet adherence, n (%)	60 (75.0%)	39 (50.0%)	< 0.001
Regular physical activity, n (%)	54 (67.5%)	35 (44.9%)	0.00
Blood glucose monitoring, n (%)	50 (62.5%)	34 (43.6%)	0.02

*Values are presented as frequencies (%). p-values are based on chi-square tests (crude comparisons)

### Harms and Unintended Effects

No intervention-related harms were identified. No adverse maternal or fetal outcomes attributable to the intervention were observed during follow-up.

## Discussion

This randomized controlled trial demonstrated that structured telephone-based self-care education significantly reduced the incidence of gestational diabetes mellitus (GDM) among high-risk pregnant women. The intervention group experienced a substantially lower GDM rate compared to controls (10.0% vs. 24.4%, *p* = 0.02), even after adjusting for potential confounders (aOR = 0.37, 95% CI: 0.15–0.88). These findings underscore the potential of low-cost telehealth interventions for effective GDM prevention, particularly in settings with limited access to specialized care.

However, reliance on self-reported adherence introduces potential reporting bias, as participants may overestimate compliance with recommended behaviors. The possibility of social-desirability bias due to self-reported adherence measures has been explicitly stated in the Limitations section of the Discussion.

### Comparison with previous research

Our results align with previous evidence suggesting that lifestyle modification initiated early in pregnancy can mitigate GDM risk ([18–19]). Similar findings were reported in the RADIEL trial by Koivusalo et al. (2016), which demonstrated that individualized lifestyle counseling before 20 weeks of gestation significantly reduced GDM incidence [16]. In contrast, smartphone-based programs such as the SMART-GDM trial [19] improved glycemic control among women with diagnosed GDM but had limited impact on weight-related outcomes. Moreover, a recent review by Raab et al. (2023) showed that the effectiveness of digital interventions depends strongly on timing, frequency, and personalization of contact [18].

However, some studies have reported conflicting or limited effects of digital interventions, particularly smartphone- or app-based tools, where evidence on improvement in pregnancy outcomes and GDM prevention remains inconsistent or non-significant ([20–21]). Our telephone-based intervention may overcome these limitations through regular, personalized contact and culturally adapted guidance, which likely enhanced participant engagement and adherence.

Our study expands upon these findings by showing that a structured telephone-based approach, implemented during the early weeks of pregnancy and integrated with routine antenatal visits, can be effective even in resource-limited healthcare systems. This preventive effect may be due to the simplicity, cultural adaptability, and regular reinforcement of lifestyle behaviors through repeated calls. Furthermore, unlike most prior studies that targeted women already diagnosed with GDM, this intervention focused on high-risk women before disease onset, demonstrating the potential value of early, preventive engagement.

Another contextual distinction is that Iranian dietary and lifestyle patterns differ substantially from Western populations. For example, high carbohydrate intake from traditional foods such as rice and date-based desserts is common, which increases the relevance of dietary education in this setting. Therefore, the stronger preventive effect observed in our trial may partly reflect the culturally tailored nature of the intervention, which incorporated practical strategies for modifying traditional meals while maintaining cultural preferences.

### Effect on weight and clinical relevance

Gestational weight control is a well-established determinant of GDM risk and maternal–fetal health. In our study, the intervention group exhibited significantly less BMI increase from baseline to 28 weeks (0.6 ± 1.1 kg/m²) compared to the control group (1.3 ± 1.4 kg/m², *p* = 0.01). Although weight control was not the primary objective, structured telephone education likely promoted healthier dietary patterns and physical activity, both of which contribute to moderated gestational weight gain. These findings are consistent with Cochrane reviews indicating that combined diet and exercise programs reduce excessive weight gain and metabolic complications when implemented early [22].

The ability of our intervention to influence weight trajectories reinforces its relevance, given that excessive GWG not only increases GDM risk but also predisposes women to long-term obesity and type 2 diabetes [23]. This supports the idea that continuous behavioral reinforcement and context-specific counseling can play an essential role in preventing both short-term and long-term metabolic disorders in pregnancy.

Behavioral and Mechanistic Considerations.

The observed improvements in adherence to key self-care behaviors—healthy diet, regular physical activity, and glucose monitoring—suggest that frequent, personalized telephone contacts serve as effective behavioral reinforcements. These improvements may also be explained by the greater frequency and continuity of contact in the intervention group, who received seven structured counseling calls, whereas the control group only attended routine in-person visits without any follow-up contact between visits. Behavior change frameworks emphasize that timely feedback and repeated engagement improve self-efficacy and sustainability of health behaviors [24].

This might explain the superiority of structured telephonic follow-up over conventional antenatal care, which often provides only episodic counseling. By maintaining consistent communication and providing culturally relevant advice, the intervention likely enhanced motivation and adherence, thereby improving physiological outcomes such as weight and glucose regulation.

Public Health and Policy Implications.

Telephone-based interventions offer unique advantages in scalability, affordability, and accessibility, especially in low- and middle-income countries where in-person lifestyle counseling may be constrained by resources. Unlike smartphone applications that require advanced digital literacy and internet connectivity, telephone counseling is widely feasible and culturally adaptable. However, despite these advantages, implementing telephone-based interventions can be time-consuming and resource-intensive, particularly when adherence is assessed through self-reported data. Ensuring consistent participation and accurate self-reporting requires regular monitoring and trained personnel, which may limit scalability in some low-resource settings. Incorporating such structured telephonic education into routine antenatal services could represent a practical, high-impact strategy to curb the growing burden of GDM [25].

Given the growing prevalence of diabetes and obesity in Middle Eastern populations, including Iran, the findings of this study highlight the importance of integrating low-cost, culturally tailored telehealth programs into the national maternal health framework. Future research should explore how these interventions can be scaled across different regions while maintaining fidelity and cultural relevance.

Approximately 94% of participants in our study reported willingness to receive health education via mobile phone, indicating a high potential acceptability for mobile-based interventions within this population.

### Discrepancies with the present study

In comparison to other studies, our study differs in several key aspects. First, while most previous research focused on lifestyle interventions in women already diagnosed with GDM, our study targeted high-risk pregnant women before the onset of the disease, which may explain the stronger preventive effects observed. This early intervention is a significant innovation in preventing GDM, as it allows for interventions before the disease manifests, potentially preventing it altogether.

Second, the telephone-based nature of our intervention, combined with cultural adaptation to Iranian dietary habits, distinguishes it from other interventions that often rely on in-person visits or smartphone-based applications. Our approach was designed to be more accessible and scalable, particularly for low-resource settings where in-person counseling may be limited.

Moreover, our study was conducted in Iran, where dietary habits and lifestyle patterns differ significantly from Western populations. For instance, high carbohydrate intake from traditional foods like rice and date-based desserts is common, making dietary education especially relevant in this setting. The culturally tailored approach of the intervention may contribute to its greater effectiveness in this context, compared to studies conducted in Western countries.

### Strengths and Limitations

The strengths of this study include its randomized design, high retention rates, and adjustment for key confounders. The inclusion of secondary outcomes such as weight changes and self-care adherence provides mechanistic insights. Although the attrition rate was relatively low (5 participants in the intervention group and 7 in the control group), it may have had a minor impact on the results. However, reliance on self-reported adherence introduces potential reporting bias, as participants may overestimate compliance with recommended behaviors. The possibility of social-desirability bias due to self-reported adherence measures has been explicitly stated in the Limitations section of the Discussion. Additionally, long-term maternal and neonatal outcomes were not assessed, warranting future studies to explore the durability of benefits and cost-effectiveness analyses across different healthcare settings.

## Conclusion

Structured telephone-based self-care education, delivered early in pregnancy to high-risk women, significantly reduced GDM incidence and attenuated gestational BMI increase compared to routine care. These findings highlight the dual benefit of this simple, low-cost, and accessible intervention in improving maternal metabolic health through both glycemic and weight-related pathways. Given its practicality, this model could be integrated into routine prenatal care programs—for example, through midwife- or nurse-led telephone counseling at primary health centers. Policymakers and maternal health planners, particularly in resource-limited settings, can consider incorporating structured telephone-based education as a standard component of antenatal care.

Future studies should assess long-term maternal and neonatal outcomes, explore cost-effectiveness at scale, and examine integration with digital health tools such as mobile apps or SMS-based follow-up systems.

## Supplementary Information

Supplementary Material 1

Supplementary Material 2

## Data Availability

The datasets used in the current study are available from the corresponding author on reasonable request.

## Abbreviations

GDM: Gestational Diabetes Mellitus
BMI: Body Mass Index
FBS: Fasting Blood Sugar
2hPP: 2-hour Postprandial Glucose
RCT: Randomized Controlled Trial
GCT: Glucose Challenge Test
OGTT: Oral Glucose Tolerance Test
aOR: Adjusted Odds Ratio
CI: Confidence Interval
MAR: Missing at Random
VIF: Variance Inflation Factor
