# Supplementary material for: Telephone-based lifestyle education to prevent gestational diabetes in high-risk Iranian women: a randomized trial
Source: BMC Pregnancy Childbirth. 2026 Feb 20;26:340. doi: 10.1186/s12884-026-08830-x (PMC13032269; doi:10.1186/s12884-026-08830-x)
Supplement: Supplementary file 1 — Supplementary Material 1 [file 12884_2026_8830_MOESM1_ESM.docx]

**Supplementary File 1: Structured Adherence Assessment Checklist for Telephone Follow-ups**

**Introduction for the Researcher:**
This checklist was developed by the research team to briefly assess participants' adherence to lifestyle recommendations during scheduled telephone follow-up calls. The questions are designed to be simple, quick to administer, and focused on key behaviors. The researcher should record the answers and provide immediate, tailored feedback and encouragement based on the responses. **(This checklist was designed for use in the scheduled telephone follow-up calls, which were conducted approximately every 10 days.)**

**Call Date:** .............
**Participant ID:** .............
**Gestational Week:** .............

**Part A: Adherence to Dietary Recommendations**

1. **Main Meals (Whole Grains):**
   - *Question:* In the past 7 days, on how many days did your main meals (breakfast, lunch, dinner) include whole-grain bread or rice?
   - *Response:* ...... days (from 0 to 7)
2. **Protein & Legume Intake:**
   - *Question:* In the past 7 days, on how many days did you include legumes (lentils, beans) or lean protein (chicken, fish) in your meals?
   - *Response:* ...... days (from 0 to 7)
3. **Healthy Snacks:**
   - *Question:* In the past 7 days, how many times did you choose fresh fruit, yogurt, or raw nuts as a snack instead of sweets or biscuits?
   - *Response:* ...... times
4. **Sugar-Sweetened Beverages:**
   - *Question:* In the past 7 days, how many times did you consume soda, industrial fruit juice, or tea with a lot of sugar?
   - *Response:* ...... times

**Part B: Adherence to Physical Activity Recommendations**

1. **Regular Physical Activity:**
   - *Question:* In the past 7 days, on how many days did you get at least 30 minutes of moderate physical activity (like brisk walking)?
   - *Response:* ...... days (from 0 to 7)
2. **Reasons for Inactivity (if applicable):**
   - *Question:* If you were unable to be active on some days, what was the main reason? (e.g., fatigue, back pain, busy schedule)
   - *Open Response:* ........................................................................................

**Part C: Adherence to Glucose Monitoring and Health Awareness**

1. **Blood Glucose Monitoring:**
   - *Question:* Did you check your blood glucose according to the agreed-upon schedule? (e.g., once or twice weekly)
   - *Response:* ◻ Yes, as planned ◻ No, less than planned ◻ Did not check at all
2. **Awareness of Warning Signs:**
   - *Question:* Have you recently experienced any symptoms like unusual thirst or hunger, frequent urination, or extreme fatigue?
   - *Response:* ◻ No ◻ Yes, mild ◻ Yes, noticeable

**Part D: General Follow-up and Support**

1. **Weight Tracking:**
   - *Question:* Are you tracking your weight at home? Is the trend following the recommended chart we discussed?
   - *Response:* ◻ Yes, it is appropriate ◻ No, it's too low/high ◻ I do not track my weight
2. **Weight Management Strategies:**
   - *Question:* What strategies did you use to manage your weight gain during this period? (e.g., controlling portion sizes, avoiding high-calorie foods)
   - *Open Response:* ........................................................................................
3. **Challenges & Successes:**
   - *Question:* What was your biggest challenge and your biggest success in following the recommendations this past period?
   - *Open Response:* ........................................................................................
4. **Need for Further Support:**
   - *Question:* Is there anything regarding the diet or exercise recommendations that you need us to explain again?
   - *Open Response:* ........................................................................................

**Researcher's Notes & Feedback:**

- *Tailored Feedback Provided:* ...........................................................................
- *Encouragement/Motivation Given:* ...................................................................
