# Supplementary material for: Telephone-based lifestyle education to prevent gestational diabetes in high-risk Iranian women: a randomized trial
Source: BMC Pregnancy Childbirth. 2026 Feb 20;26:340. doi: 10.1186/s12884-026-08830-x (PMC13032269; doi:10.1186/s12884-026-08830-x)
Supplement: Supplementary file 2 — Supplementary Material 2 [file 12884_2026_8830_MOESM2_ESM.docx]

**CONSORT 2025 Checklist**

| **Section/Topic** | **Item No** | **Checklist item** | **Reported on section/page** |
| --- | --- | --- | --- |
| **Title and abstract** | 1a | Identification as a randomised trial in the title | Title page ( "Telephone-Based Lifestyle Education to Prevent Gestational Diabetes in High-Risk Iranian Women: A Randomized Trial")( Page 1) |
| **Title and abstract** | 1b | Structured summary of trial design, methods, results, and conclusions | Abstract (Page 2) |
| **Open Science** | 2 | Name of trial registry, identifying number (with URL) and date of registration | Methods: Study Design and Setting (IRCT under IRCT2017052115995N2) (Page 3) |
| **Open Science** | 3 | Where the trial protocol and statistical analysis plan can be accessed | Methods (Pages 5-10) |
| **Open Science** | 4 | Where and how the individual de-identified participant data (including data dictionary), statistical code, and any other materials can be accessed | Availability of data and materials (Page 20) |
| **Open Science** | 5a | Sources of funding and other support (e.g., supply of drugs), role of funders | Funding (Page 20) |
| **Open Science** | 5b | Financial and other conflicts of interest of the manuscript authors | Competing interests (Page 20) |
| **Introduction** | 6 | Scientific background and explanation of rationale | Background (Page 3) |
| **Introduction** | 7 | Specific objectives or hypotheses related to benefits and harms | Background (Page 3-4) |
| **Methods** | 8 | Details of patient or public involvement in the design, conduct, and reporting of the trial | Study Design and Setting (Page 4) |
| **Methods** | 9 | Description of trial design (such as parallel, factorial) including allocation ratio, and framework (e.g., superiority) | Methods: Study Design and Setting (parallel-group RCT, 1:1 ratio) (Page 6) |
| **Methods** | 10 | Important changes to methods after trial commencement (such as eligibility criteria), with reasons | Study Design and Setting (Page 4) |
| **Methods** | 11 | Settings and locations where the data were collected | Methods: Study Design and Setting (25 urban and rural primary health care centers, Abadan University) (Page 4) |
| **Methods** | 12a | Eligibility criteria for participants | Methods: Inclusion and Exclusion Criteria |
| **Methods** | 12b | If applicable, eligibility criteria for sites and individuals delivering interventions | Methods: Intervention |
| **Methods** | 13 | The interventions for each group with sufficient details to allow replication, including how and when they were actually administered | Methods: Intervention (detailed sessions, Table 1) |
| **Methods** | 14 | Completely defined pre-specified primary and secondary outcome measures, including how and when they were assessed | Methods: Outcome Measures (GDM incidence at 28 weeks, BMI change, adherence) |
| **Methods** | 15 | How harms were defined and assessed (e.g., systematically, non-systematically) | **Harms and Unintended Effects**  (Page 15) |
| **Methods** | 16 | How sample size was determined | Methods: Participants and Screening (calculated based on effect size 0.5, n=63 per group, recruited 170) |
| **Methods** | 17a | Method used to generate the random allocation sequence | Methods: Randomization and Allocation (computer-generated sequence) |
| **Methods** | 17b | Type of randomisation; details of any restriction (such as blocking and block size) | Methods: Randomization and Allocation (block randomization, block size 4, stratified by education and BMI) |
| **Methods** | 18 | Mechanism used to implement the random allocation sequence (such as sequentially numbered containers), describing any steps taken to conceal the sequence until interventions were assigned | Methods: Randomization and Allocation (Page 5) |
| **Methods** | 19 | Who generated the random allocation sequence, who enrolled participants, and who assigned participants to interventions | Methods: Randomization and Allocation (independent statistician generated, trained staff enrolled) |
| **Methods** | 20a | If done, who was blinded after assignment to interventions (for example, participants, care providers, those assessing outcomes) and how | Randomization and Allocation (Page 4) |
| **Methods** | 20b | If relevant, description of the similarity of interventions | N/A |
| **Methods** | 21a | Statistical methods used to compare groups for primary and secondary outcomes | Methods: Statistical Analysis (chi-square, t-tests, logistic regression) |
| **Methods** | 21b | Methods for additional analyses, such as subgroup analyses and adjusted analyses | Methods: Statistical Analysis |
| **Results** | 22a | For each group, the numbers of participants who were randomly assigned, received intended treatment, and were analysed for the primary outcome | Results: Participant Flow (n=85 each, completed 80 and 78) |
| **Results** | 22b | For each group, losses and exclusions after randomisation, together with reasons | Results: Participant Flow (5 lost in intervention, 7 in control, reasons given) |
| **Results** | 23 | Dates defining the periods of recruitment and follow-up | Methods: Study Design (October 2024 to June 2025) |
| **Results** | 24 | A table showing baseline demographic and clinical characteristics for each group | Results: Table 2a and 2b |
| **Results** | 25a | For each group, number of participants (denominator) included in each analysis and whether the analysis was by original assigned groups | Results: Primary Outcome (n=80 and 78, ITT mentioned) |
| **Results** | 25b | For each primary and secondary outcome, results for each group, and the estimated effect size and its precision (such as 95% confidence interval) | Results: Table 3, 4, 5 (aOR = 0.37, 95% CI: 0.15–0.88) |
| **Results** | 26 | Results of any other analyses performed, including subgroup analyses and adjusted analyses, distinguishing pre-specified from exploratory | Results: Multivariable logistic regression |
| **Results** | 27 | All important harms or unintended effects in each group (for specific guidance see CONSORT for harms) | **Harms and Unintended Effects**  (Page 15) |
| **Discussion** | 28 | Trial limitations, addressing sources of potential bias, imprecision, and, if relevant, multiplicity of analyses | Discussion: Strengths and Limitations |
| **Discussion** | 29 | Generalisability (external validity, applicability) of the trial findings | Discussion: Public Health and Policy Implications |
| **Discussion** | 30 | Interpretation consistent with results, balancing benefits and harms, and considering other relevant evidence | Discussion: Comparison with Previous Research, Conclusion |
